# Regulation of mitochondrial permeability transition pore by PINK1

**DOI:** 10.1186/1750-1326-7-22

**Published:** 2012-05-25

**Authors:** Emilie Giaime, Erica Caballero, Lucía Núñez, Zhiyin Song, David Chan, Carlos Villalobos, Jie Shen

**Affiliations:** 1 Center for Neurologic Diseases, Department of Neurology, Brigham & Women’s Hospital, Program in Neuroscience, Harvard Medical School, Boston, MA, 02115, USA; 2 Instituto de Biología y Genética Molecular, Universidad de Valladolid and Consejo Superior de Investigaciones Científicas, Valladolid, Spain; 3 Division of Biology and Howard Hughes Medical Institute, California Institute of Technology, Pasadena, CA, 91125, USA; 4 Université Pierre et Marie Curie-Paris 6, Centre de Recherche de l'Institut du Cerveau et de la Moelle épinière, UMR-S975, Paris, 75013, France

**Keywords:** Parkinson’s disease, Mitochondrial respiration, Mitochondrial transmembrane potential, Mitochondrial permeability transition pore, Calcium

## Abstract

**Background:**

Loss-of-function mutations in PTEN-induced kinase 1 (PINK1) have been linked to familial Parkinson’s disease, but the underlying pathogenic mechanism remains unclear. We previously reported that loss of PINK1 impairs mitochondrial respiratory activity in mouse brains.

**Results:**

In this study, we investigate how loss of PINK1 impairs mitochondrial respiration using cultured primary fibroblasts and neurons. We found that intact mitochondria in *PINK1*−/− cells recapitulate the respiratory defect in isolated mitochondria from *PINK1*−/− mouse brains, suggesting that these *PINK1*−/− cells are a valid experimental system to study the underlying mechanisms. Enzymatic activities of the electron transport system complexes are normal in *PINK1*−/− cells, but mitochondrial transmembrane potential is reduced. Interestingly, the opening of the mitochondrial permeability transition pore (mPTP) is increased in *PINK1*−/− cells, and this genotypic difference between *PINK1*−/− and control cells is eliminated by agonists or inhibitors of the mPTP. Furthermore, inhibition of mPTP opening rescues the defects in transmembrane potential and respiration in *PINK1*−/− cells. Consistent with our earlier findings in mouse brains, mitochondrial morphology is similar between *PINK1*−/− and wild-type cells, indicating that the observed mitochondrial functional defects are not due to morphological changes. Following FCCP treatment, calcium increases in the cytosol are higher in *PINK1−/−* compared to wild-type cells, suggesting that intra-mitochondrial calcium concentration is higher in the absence of PINK1.

**Conclusions:**

Our findings show that loss of PINK1 causes selective increases in mPTP opening and mitochondrial calcium, and that the excessive mPTP opening may underlie the mitochondrial functional defects observed in *PINK1*−/− cells.

## Introduction

Recessively inherited loss of function mutations in the *PINK1* gene have been linked to familial Parkinson’s disease (PD). The PINK1 protein bears a 34 amino acid mitochondrial targeting domain [1] and has been shown to localize within mitochondria [2]. Mitochondrial dysfunction has long been thought to play a key role in PD pathogenesis, based in part on postmortem studies that showed mitochondrial impairment (*e.g.* reduced complex I activity) and oxidative damage in idiopathic PD brains [3]. This is further supported by observations that mitochondrial complex I inhibitors, such as MPTP [4] and rotenone [5] produce parkinsonian syndromes in humans and experimental animal models.

Genetic studies in *Drosophila* showed that PINK1 is involved in the maintenance of mitochondrial morphology by interacting with components of the mitochondrial fission and fusion machinery [6-9]. Loss of PINK1 in *Drosophila* appears to promote mitochondrial fusion, though the effects of PINK1 inactivation on mitochondrial morphology in cultured mammalian cells are less consistent, ranging from promotion of mitochondrial fragmentation or fusion to no effects [10-14]. Despite the controversial findings on the effects of PINK1 inactivation on mitochondrial morphology in mammalian culture systems, several functional defects have been reported consistently, including impairment of mitochondrial respiration [15-20] and reduction of mitochondrial transmembrane potential [1,11,15,16,21]. Our previous analysis of *PINK1*−/− mice led to the first report showing that PINK1 is required for mitochondrial respiratory function *in vivo*[18]. However the cause of these functional defects remains to be elucidated.

To determine the pathogenic cascade of events in intact mitochondria, we derived primary mouse embryonic fibroblasts (MEFs) and cortical neuronal cultures from our *PINK1*−/− mice. Similarly to what we previously reported in isolated mitochondria from the brain, mitochondrial respiration is impaired in *PINK1*−/− cells. While the enzymatic activity of each complex composing the electron transport system is normal, mitochondrial transmembrane potential (ΔΨ_m_) is reduced in *PINK1*−/− MEFs and neurons. Interestingly, the reduction of ΔΨ_m_ in *PINK1*−/− cells is associated with increased opening of the mitochondrial permeability transition pore (mPTP). Inhibition of the mPTP reverses the depolarization of the mitochondrial inner membrane and respiration defects seen in *PINK1*−/− cells. We did not find evidences of increased oxidative stress, a common inducer of mPTP opening. In addition, we found no detectable changes in mitochondrial morphology in *PINK1*−/− cells. Together our findings highlight a role of PINK1 in the regulation of the mitochondrial permeability transition pore and suggest that increased opening of the pore in the absence of PINK1 may be responsible for the reduced mitochondrial transmembrane potential and the reduced respiratory activities.

## Materials and methods

### Primary MEF and cortical cultures

Mouse embryonic fibroblasts (MEFs) were derived from embryos at embryonic day 14.5. After removing the head and the inner organs embryos were individually minced with scissors, treated with trypsin (1% v/v) for twelve minutes at 37°C and dispersed mechanically and plated with MEF media (Dulbecco′s Modified Eagle Medium (DMEM), 10% fetal bovine serum (FBS), penicillin/streptomycin (Gibco, Life Technologies, Grand Island, NY, USA)). After they reached ~100% confluency, cells were frozen down in DMEM containing 10% DMSO (Sigma, St Louis, MO, USA). The number of MEF samples used in each experiment is specified in the figure legends and reflects the number of individual cultures derived from individual embryos used to derive MEFs.

Primary cortical cultures were prepared and maintained as described previously [22]. Experiments were performed at 14 ± 1 days *in vitro*. Cortices from different pups were not pooled, and the number of experiments specified in the legend reflects the number of different cultures derived from individual pups.

### Mitochondrial respiration

Mitochondrial respiration was assayed as the O_2_ consumption of cell suspension using a Clark electrode (Rank Brothers Ltd, Cambridge, England). Cells were resuspended to a final density of 2.10^6^ cells/ml in respiration buffer (0.137 M NaCl, 5 mM KCl, 0.7 mM NaH_2_PO_4_, 25 mM Tris, pH 7.4 at 25°C). Endogenous respiration activity was measured after addition of glucose (10 mM, Sigma). For complex driven respiration, plasma membranes were permeabilized by addition of digitonin at a final concentration of 0.01% (Sigma). Cells were supplemented with substrates for either complex I (10 mM glutamate/malate, Sigma), II (10 mM succinate, Sigma) or III (1 mM TMPD/1 mM ascorbate, Sigma) together with adenosine diphosphate (ADP, 1 mM, Sigma) to the recording chamber. State 3 respiration activity was then measured. ADP independent respiration activity (State 4) was monitored after addition of oligomycin (2 μM, Sigma).

### Enzymatic activity of ETS complexes and ATP synthase

All assays were performed on mitochondria isolated from MEFs according to a previously established method [23]. For each complex 5 μg of mitochondrial proteins and 100 μl of each assay buffer were used. Complex I (NADH: ubiquinone oxidoreductase) buffer (35 mM NaH_2_PO_4_ pH 7.2, 5 mM MgCl_2_, 0.25% BSA, 2 mM KCN, 1 μM antimycin, 97.5 μM ubiquinone-1, 0.13 mM NADH, Sigma). Only the rotenone sensitive activity was monitored by following the oxidation of NADH at 340 nm (OD 6220 M^-1^.cm^-1^). Complex II (succinate dehydrogenase) buffer (25 mM KH_2_PO_4_, 5 mM MgCl_2_, pH 7.2, 20 mM succinate, 50 μM 2,6-dichlorophenolindophenol (DCPIP), 0.25% BSA, 2 mM KCN, 1 μM antimycin, Sigma). Enzymatic activity was monitored by the reduction of DCPIP/PES at 600 nm (OD 19100 M^-1^.cm^-1^) after addition of 65 μM ubiquinone 1. Complex III activity (decylubiquinol/ferricytochrome C oxidoreductase) buffer (3 mM sodium azide, 1.5 μM rotenone, 50 μM ferricytochrome C, and 50 mM phosphate buffer, pH 7.2, Sigma). Reaction was followed as the increase in reduced Cytochrome C absorbance at 550 nm (OD 18500 M^-1^.cm^-1^) after the addition of 35 μM of freshly prepared ubiquinol 2. Complex IV (Cytochrome C oxidase) activity and Complex II + III (succinate-Cytochrome C reductase) activities were previously described [18]. Levels of Cytochrome C were measured by western blot using a commercial antibody (Cell signalling Technology, Danvers, MA, USA).

### Measurement of mitochondrial transmembrane potential and mPTP opening

Mitochondrial ΔΨ was measured with the non-quenching Tetramethylrhodamine, methyl ester (TMRM) fluorescence methods (Molecular Probes, Life Technologies). MEFs were stained with TMRM (50 nM) in DMEM for 30 min at 37°C in the dark. The cells were then washed twice with PBS. Mitochondrial PTP opening was assessed by the quenching of calcein-AM fluorescence by cobalt [24]. Thirty min after cells were loaded with Calcein-AM (1 μM, Molecular Probes, Life Technologies) at 37°C in the dark, CoCl_2_ (1 mM, Sigma) was added and cells incubated for another 10 min. Then, fluorescence of 30,000 cells for each experiment was measured with a flow cytometer (FACSCalibur), and the data were processed with the CellQuest program (BD Biosciences, San Jose, CA, USA). Neurons were incubated for 30 min with TMRM (50 nM) in neuronal extracellular buffer with calcium or with Calcein (1 μM) for 45 min in the dark after 30 min CoCl_2_ (1 mM) was added. Then cells were washed and imaged on a Leica DMI6000 Microscope. Imaging processing and data analysis were performed using LASAF software (Leica, Wetzlar, Germany). In some experiments cells were pre-incubated for 1 hr with atractylate (20 μM, Sigma), Cyclosporine A (CsA, 1 μM, Sigma), Bongkrekic acid (BkA, 10 μM, Sigma), FK-506 (5 μM, Sigma), 0.1% vehicle (DMSO), Tocopherol (50 μM, 4 hr, Sigma) or NAC (1 mM, 2 hr, Sigma).

For imaging expreriments, MEFs were cultured on glass bottom culture dishes. Cells were loaded for Δψ_m_ with TMRM (50 nM) and Mitotracker Green (200 nM) (Molecular Probes, Life Technologies) with or without Oligomycin (1 μM), FCCP (1 μM) or CoCl_2_ (1 mM). For the mPTP opening assay, cells were loaded with calcein-AM (1 μM) and Mitotracker Red (150 nM) (Molecular Probes, Life Technologies), with or without CoCl_2_ (1 mM) both in HBSS 1X (Gibco, Life Technologies) for 20 min at 37°C. Then, cells were washed three times in HBSS 1X. Live images of the cells were captured with the Olympus FluoView FV1000 Confocal Microscope (Olympus Imaging America Inc, Center Valley, PA, USA) and analyzed using ImageJ software.

### Oxidative stress assay

To measure H_2_O_2_ production mitochondria were isolated using the mitochondrial isolation kit from Sigma according to the manufacturer instructions. The experiment was started by adding 100 μl of assay buffer (HBSS containing 10 μM Amplex Red, 10 mM succinate, 0.2 units/ml Horse Radish Peroxidase) and followed over time on a fluorescence plate reader. The same conditions were used to determine the production of superoxide anion using the Dihydroethidium (10 μM, DHEt) method. The protein carbonyl contents in cell lysates were detected by the OxyBlot protein oxidation detection kit (Millipore, Billerica, MA) using the instructions provided by the manufacturer. Lipid peroxidation was analyzed using the ThioBarbituric Acid Reactive Species (TBARS) assay and according to the manufacturer's instructions (Cayman Chemical, Ann Arbor, MI, USA).

### Analysis of mitochondrial morphology

For visualization of mitochondria, MEFs were either stained with Mitotracker Red (250 nM) or infected with a retrovirus expressing mt-DsRed [25]. Primary cultured neurons were only imaged with MitoTracker Red (100 nM). Then cells were washed for 10 min and fixed with 4% PFA (Electron Microscopy Science, Hatfield, PA, USA) for 20 min. After fixation coverslips were mounted on glass slides and imaged by epifluorescence on a Leica DMI6000 Microscope (Leica Microsystems GmbH, Wetzlar, Germany). For live imaging, after staining cells were mounted on a perfusion chamber in culture media containing HEPES (1 mM) and imaged at 22°C. Regardless of the staining method, cells were then scored by eye into four different categories according to the morphology of their mitochondrial network previously described [25]. The automatic analysis of the size and branching of the mitochondrial network was done using the particle analysis function of ImageJ according to a previously described methods using ImageJ [26].

### Calcium imaging

FCCP releasable pool was measured by adapting a previously described method [27]. Briefly, MEFs and primary cortical neurons cultures were loaded with Fura-2 AM (5 μM, 45 min at 37°C) (Molecular probes, Life Technologies), and imaged with a Leica DMI6000 Microscope. Imaging processing and data analysis were performed using LAS AF software (Leica). FCCP (1 μM) was applied using an 8-channel gravity perfusion system (ALA Scientific Instrument, Farmingdale, NY, USA).

### Statistical analysis

Statistical analysis was performed using Prism 5 (Graph-Pad Software) and Excel (Microsoft). Pooled results were expressed as means ± SEM. Significance was determined by the non paired Student *t*-test.

## Results

### Respiratory defects in intact mitochondria but normal activities of enzymes in the mitochondrial electron transport system (ETS) in *PINK1*-deficient cells

**Figure 1:**
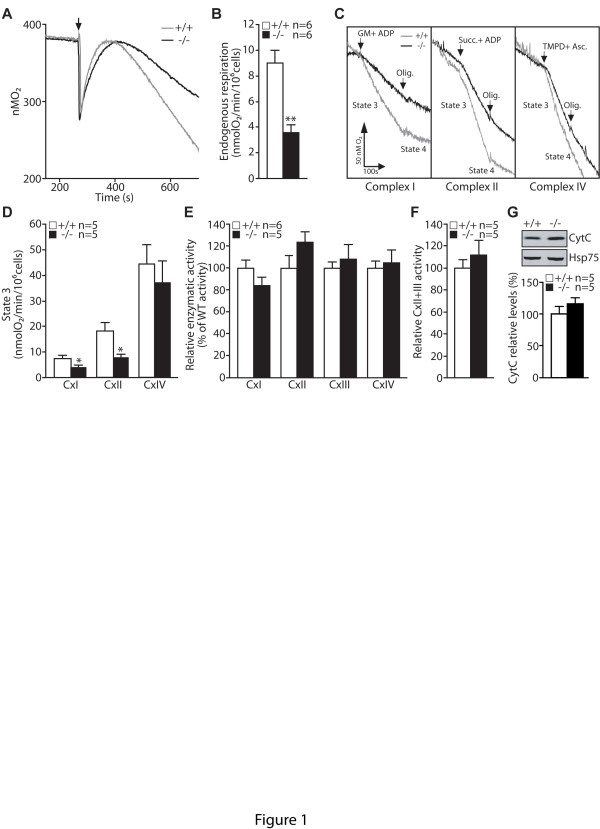
**Impaired mitochondrial respiration in*PINK1*−/− fibroblasts. A**. Representative oxygraphs of *PINK1*−/− (−/−) and wild-type (+/+) MEFs energized with glucose (10 mM). The arrow indicates the time MEFs are added to the chamber. **B**. Bars show oxygen consumption, which represents the endogenous respiratory activity in *PINK1*−/− and +/+ MEFs. **C** Representative oxygraphs of *PINK1*−/− and +/+ MEFs energized with 10 mM glutamate/malate (complex I substrate), 10 mM succinate (complex II substrate) or 1 mM TMPD/1 mM ascorbate (complex IV substrate) in the presence of ADP (1 mM). Arrows indicate the time of the addition of either the substrates or oligomycin (2 μM). **D**. Bars show State 3 respiratory activity for complex I, II and IV in *PINK1*−/− and +/+ MEFs permeabilized with digitonin. **E**. Enzymatic activities of complexes I, II, III and IV of the mitochondrial electron transport system, as measured by spectrophotometric assays and after normalization to citrate synthase activity. **F**. Graph represents succinate-cytochrome C oxidase (complex II + III) activity. **G**. Upper panel: Representative western blot showing levels of cytochrome C in the mitochondrial fraction of *PINK1*−/− and *+/+* MEFs. Hsp75 is used as a control for the total amount of mitochondrial proteins loaded in each well. Lower panel: The bar graph shows relative quantification of the level of Cytochrome C using Hsp75 as loading control. All data are expressed as mean ± SEM. * *p* < 0.05, ** *p* < 0.01.

Our previous study revealed that respiratory activity is impaired in mitochondria isolated from striata or aged cortices of *PINK1*−/− mice [18]. To investigate further the underlying mechanisms, we derived mouse embryonic fibroblasts (MEFs) from *PINK1*−/− mice. MEFs allow the functional investigation of mitochondria in an intact cellular environment and are more amenable to experimental manipulations than mitochondria isolated from brains. We first examined whether intact mitochondria in *PINK1*−/− MEFs recapitulate the mitochondrial defects observed in isolated mitochondria from *PINK1*−/− striata. We measured the endogenous respiratory activity of primary MEFs energized with glucose (10 mM). Consistent with our earlier *in vivo* findings, endogenous respiration rate is reduced in *PINK1*−/− fibroblasts (Figure 1A and 1B).

Respiratory defects in *PINK1*-deficient cells have been suggested to result from impaired plasma membrane glucose transporter activity, which reduces substrates delivery to mitochondria [15]. To bypass the glucose transporter, we measured the respiratory activity of digitonin-permeabilized cells energized with 10 mM glutamate/malate (complex I substrate) or 10 mM succinate (complex II substrate) or 1 mM Ascorbate/1 mM TMPD (complex IV substrates) in the presence of saturating concentrations of ADP (1 mM). The use of digitonin (0.01% final concentration) allows the direct delivery of substrates to mitochondria by specifically permeabilizing the plasma membrane without affecting mitochondrial integrity. Interestingly, we found that state 3 activities, which represent the maximum respiration rate in the presence of ADP are significantly decreased for complex I and complex II substrates in *PINK1*−/− fibroblasts (Figure 1C and 1D). These results are similar to what we and others have independently reported in *PINK1*−/− mice and MEFs [16,18], indicating that *PINK1*−/− MEFs represent a valid cellular model to study the detailed mechanisms underlying respiratory defects seen in *PINK1*−/− mice.

Oxidative phosphorylation is a complex process relying on the proper function of several enzymatic complexes and the maintenance of transmembrane potential (ΔΨ_m_). We therefore measured the enzymatic activities of all individual complexes composing the electron transport system (ETS) in MEFs. Using spectrophotometric methods, we measured enzymatic activities for complex I (NADH-ubiquinone reductase activity), complex II (succinate-ubiquinone reductase activity), complex III (ubiquinol-Cytochrome C reductase activity) and complex IV activity (Cytochrome oxidase activity). After normalization to citrate synthase activity, enzymatic activities of all complexes composing the ETS appear normal in *PINK1*−/− MEFs (Figure 1E). Reduced respiration has been documented to result from coenzyme Q deficiency, which affects the transfer of electrons from complexes I or II to III [28]. We investigated a possible coenzyme Q deficiency by measuring the antimycin sensitive succinate-Cytochrome C reductase activity. This activity was also found normal in *PINK1*−/− MEFs (Figure 1F). Reduced Cytochrome C in the intermembrane space results in reduced electron transfer between complex III and IV and impairs mitochondrial respiratory activities. We measured the level of Cytochrome C in the mitochondrial fraction of *PINK1*−/− MEFs and control cells and found that the levels were similar between the two genotypic groups after normalization using Hsp75 as loading control (Figure 1G).

### Reduced mitochondrial transmembrane potential of *PINK1*−/− MEFs

**Figure 2:**
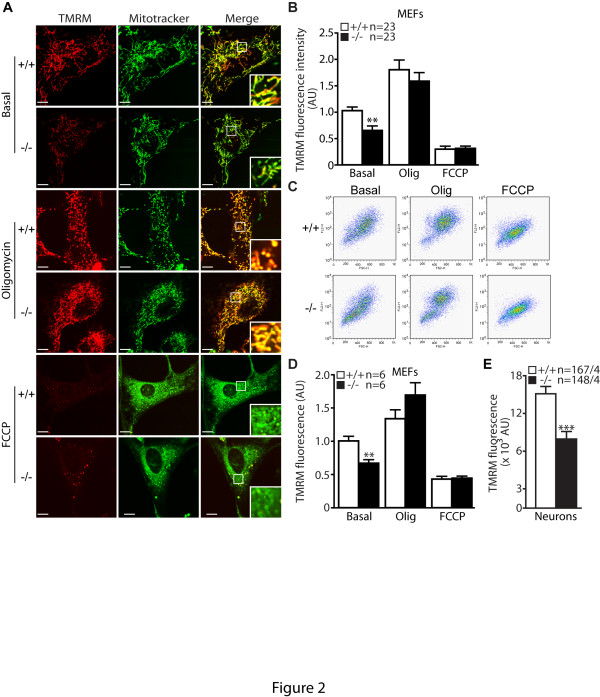
**Reduced mitochondrial transmembrane potential in*PINK1*−/− cells. A**. Representative confocal microscopic images of *PINK1*−/− and +/+ MEFs after staining with TMRM (50 nM, red) and Mitotracker Green (200 nM) in the presence or absence of oligomycin (Olig, 1 μM) or FCCP (10 μM). The intensity of TMRM reflects the level of mitochondrial transmembrane potential, whereas the intensity of Mitotracker Green is not affected by transmembrane potential. The bottom right inserts are the higher power views of the boxed areas in the same panel. The TMRM signal is reduced in *PINK1*−/− cells, whereas the TMRM signal is increased and decreased similarly in both *PINK1*−/− and +/+ cells following oligomycin and FCCP treatment, respectively. **B**. The bar graph shows quantification of TMRM signals in *PINK1*−/− and +/+ MEFs in the presence or absence of oligomycin and FCCP using confocal images. The number shown in the panel indicates the number of cells used in the study. **C**. Representative flow cytometry dot plots show the intensity of TMRM signals in *PINK1*−/− and +/+ MEFs following incubation with TMRM (50 nM) in the presence or absence of oligomycin (1 μM) or FCCP (10 μM). **D**. The bar graph shows quantification of TMRM signals measured by FACS analysis in *PINK1*−/− and +/+ MEFs. The number shown in the panel indicates the number of independent experiments performed. **E**. The bar graph of TMRM fluorescent signals in *PINK1*−/− and +/+ neurons shows reduced TMRM signals in *PINK1*−/− neurons. The numbers shown indicate the number of neurons used (left) and the number of independent experiments performed (right) in the study. All data are expressed as mean ± SEM. Scale bar: 10 μm. * *p* < 0.05, ** *p* < 0.01, *** *p* < 0.001.

In the absence of enzymatic defects of the ETS complexes, we turned our attention towards mitochondrial transmembrane potential (ΔΨ_m_), the electrochemical force that modulates the kinetics of proton reentry to the matrix through ATP-synthase. Using microscopic and flow cytometric analyses, we measured the transmembrane potential of MEFs stained with TMRM (50 nM). TMRM is a cationic fluorescent dye that accumulates inside the mitochondrial matrix according to the membrane potential. Interestingly, TMRM fluorescence signal is reduced in both experiments in *PINK1*−/− MEFs (Figure 2A-D). To ensure that dye is equally loaded and that the TMRM signal is not auto-quenched we compared TMRM fluorescence in *PINK1*−/− and control MEFs following oligomycin and FCCP treatment. Oligomycin, an inhibitor of ATP synthase, induces hyperpolarization of mitochondria and increases of TMRM fluorescence, whereas FCCP dissipates transmembrane potential. No differences in TMRM fluorescence between the two genotypes were found following either of these treatments.

We investigated whether ΔΨ_m_ is similarly affected by the loss of PINK1 in primary cortical neurons. Because flow cytometry requires re-suspension of cells that could damage mature neurons, we measured ΔΨ_m_ using fluorescence microscopy. Neuronal cultures were stained with TMRM (50nM), imaged with a fluorescent microscope and the intensity of the TMRM fluorescence was determined for each individual neuron. Consistent with what we have observed in fibroblasts, TMRM fluorescence is reduced by 40% in *PINK1*−/− neurons, confirming a reduction of ΔΨ_m_ in the absence of PINK1 (Figure 2E).

### Increased mPTP opening in the absence of PINK1

Since the enzymatic activity of each component of the ETS is normal, we looked for alternative mechanisms underlying the reduction of ΔΨ_m_. We evaluated opening of the mitochondrial permeability transition pore, which allows the diffusion of small ions across the mitochondrial inner membrane [29]. We compared opening of mPTP in *PINK1*−/− and control MEFs under basal conditions using the CoCl_2_-calcein fluorescence-quenching assay [30]. Calcein-AM is a membrane permeable fluorophore that diffuses freely into all subcellular compartments including mitochondria. The acetoxymethyl (AM) group of the fluorophore is cleaved by ubiquitous intracellular esterase. Calcein, which is hydrophilic, is then trapped within all subcellular compartments. The cells are then loaded with the divalent cobalt cation (Co^2+^), which quenches calcein fluorescence in all subcellular compartments except the mitochondrial matrix, as the inner mitochondrial membrane is the only intracellular membrane that is normally Co^2+^-impermeable [30,31]. However, during the opening of the mPTP, cobalt enters mitochondria and is able to quench mitochondrial calcein fluorescence [32].

**Figure 3:**
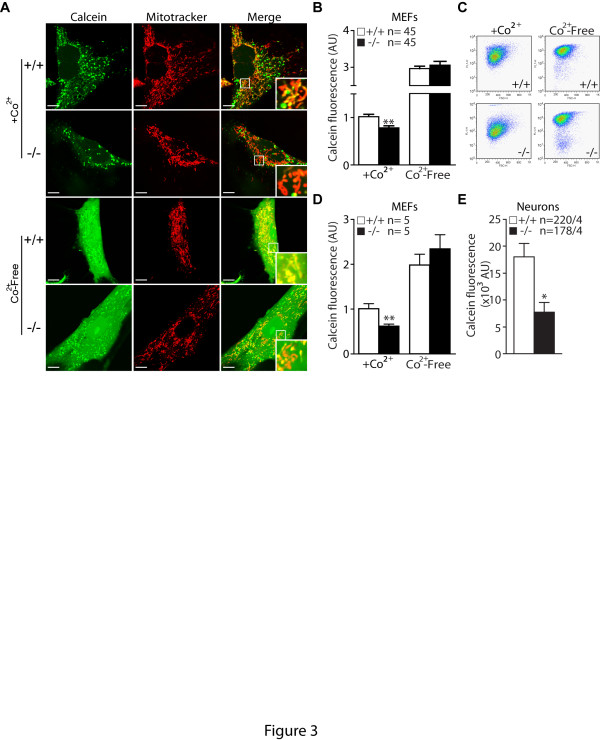
**Increased opening of the mitochondrial permeability transition pore in*PINK1*−/− cells. A**. Representative confocal microscopic images of *PINK1*−/− and +/+ MEFs after incubation with calcein-AM (1 μM) and Mitotracker Deep Red (150 nM) in the presence or absence of Co^2+^ (1 mM), which quenches calcein fluorescence (green) outside of mitochondria. Mitotracker Deep Red allows visualization of calcein fluorescence in mitochondria. The bottom right inserts are the higher power views of the boxed areas in the same panel. The calcein fluorescence in mitochondria is lower in *PINK1*−/− cells in the presence of Co^2+^. In the absence of Co^2+^, calcein fluorescent signals are very intense and are present in the entire cell, and there are no genotypic differences. **B**. The bar graph shows quantification of calcein fluorescence in *PINK1*−/− and +/+ cells in the presence or absence of Co^2+^ using confocal images. The number shown in the panel indicates the number of cells used in the study. **C**. Representative flow cytometry dot plots show the intensity of calcein signals in *PINK1*−/− and +/+ MEFs following incubation with calcein-AM (1 μM) in the presence or absence of Co^2+^ (1 mM). **D**. The bar graph of calcein signals measured by FACS analysis shows reduced calcein signals in *PINK1*−/− MEFs in the presence of Co^2+^. The number shown in the panel indicates the number of independent experiments performed. **E**. The bar graph of calcein fluorescent signals in *PINK1*−/− and +/+ neurons shows reduced calcein signals in *PINK1*−/− neurons. The numbers shown indicate the number of neurons used (left) and the number of independent experiments performed (right) in the study. All data are expressed as mean ± SEM. Scale bar: 10 μm. * *p* < 0.05, ** *p* < 0.01.

Under basal conditions, calcein fluorescence measured by both microscopic and flow cytometric analyses is lower in *PINK1*−/− MEFs, suggesting increases in mPTP opening (Figure 3A-D). We also measured calcein fluorescence in the absence of cobalt. As expected, calcein fluorescence is much higher in the absence of Co^2+^ and is similar between *PINK1*−/− and control cells, indicating similar calcein loading (Figure 3A-D). We extended the analysis to *PINK1*−/− neurons to confirm if mPTP opening is also increased. Because flow cytometric analysis requires re-suspension of cultured cells and thus would damage mature neurons, we used only microscopic analysis [33]. We found marked reduction of calcein fluorescence in *PINK1*−/− cortical neurons, further confirming increases in mPTP opening in the absence of PINK1 (Figure 3E).

**Figure 4:**
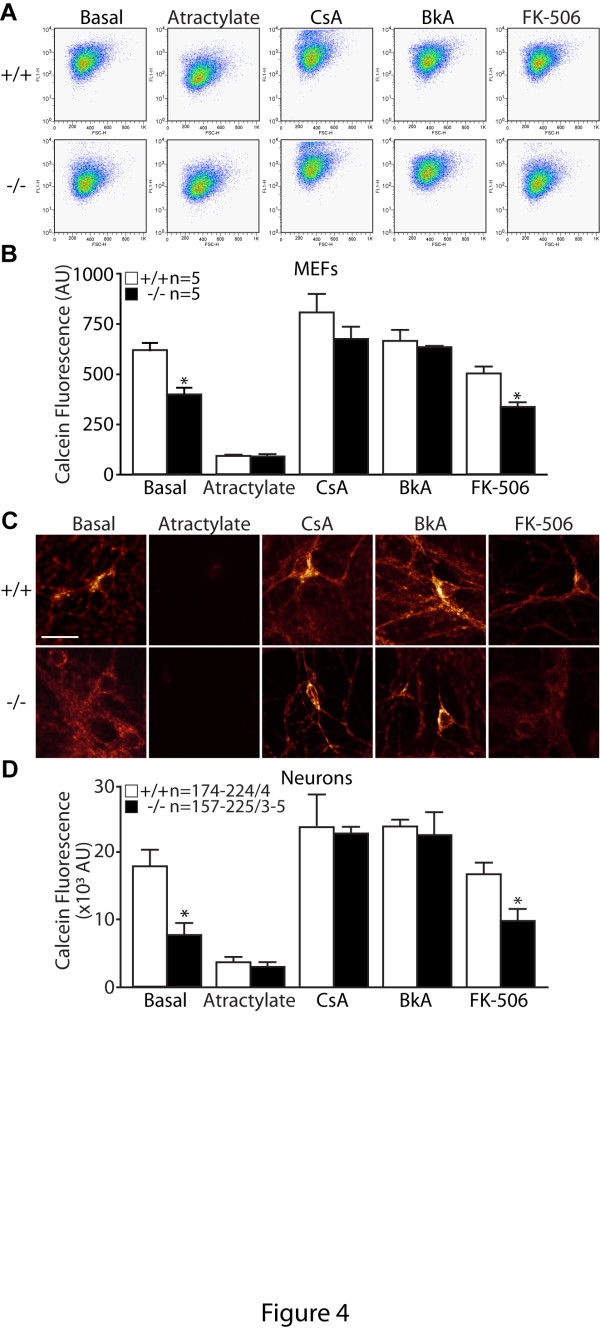
**Effects of mPTP agonists and inhibitors on its opening in*PINK1*−/− cells. A**. Representative flow cytometry dot plots showing calcein fluorescence in *PINK1*−/− and +/+ cells treated with calcein-AM (1 μM) and Co^2+^ (1 mM) under basal conditions or following various treatment, such as in presence of the mPTP agonist atractylate (20 μM), the mPTP inhibitors Cyclosporine A (CsA, 1 μM) or Bongkrekic acid (BkA, 10 μM), or the calcineurin inhibitor FK-506 (5 μM). **B**. The bar graph shows quantification of calcein fluorescence after FACS analysis following various treatment, such as atractylate (20 μM), CsA (1 μM), BkA (10 μM), or FK-506 (5 μM). The number shown in the panel indicates the number of independent experiments performed. **C**. Representative images showing calcein fluorescence in *PINK1*−/− and +/+ cortical neurons incubated with calcein-AM (1 μM) and Co^2+^ (1 mM) under basal conditions or in the presence of atractylate (20 μM), CsA (1 μM), BkA (10 μM) or FK-506 (5 μM). **D**. Quantification of calcein fluorescence in *PINK1*−/− and +/+ neurons following various treatment with atractylate (20 μM), CsA (1 μM) or BkA (10 μM) or FK-506 (5 μM). The numbers shown in panels indicate the number of cells used (left) and the number of independent experiments performed (right) in the study. All data are expressed as mean ± SEM. * *p* < 0.05.

We further tested whether treatment of atractylate, an agonist of the mPTP, would result in decreases of calcein fluorescence. Following the treatment, calcein signal is considerably reduced and the low level of calcein fluorescence is similar between the two genotypic groups in both MEFs and neurons (Figure 4A-D). These results provide additional support that the lower calcein signal in *PINK1*−/− cells is due to increased opening of the mPTP.

We further treated cells with two agents that can inhibit opening of the mPTP. Treatment with cyclosporin A (CsA), an inhibitor of mPTP, eliminated the genotypic difference in calcein fluorescence between *PINK1*−/− and control MEFs and neurons (Figure 4). Although CsA is a strong inhibitor of the mPTP [34], it has additional molecular targets such as calcineurin [35]. We therefore used another mPTP inhibitor, bongkrekic acid, which inhibits the mitochondrial ATP-ADP translocase, a component of the mPTP, and has no inhibitory effect on calcineurin [36]. Treatment with bongkrekic acid also abrogates the genotypic difference in MEFs and neurons (Figure 4). Furthermore, treatment of cells with FK-506, a potent calcineurin inhibitor that has no direct effect on the mPTP [37], showed that it has little effect on calcein signals (Figure 4). Using fluorescence based enzymatic assay we confirmed that treatment with FK-506 (5 μM) results in an almost complete inhibition of calcineurin activity (data not shown). These results provide further support that loss of PINK1 results in increased opening of the mPTP.

### Inhibition of the mPTP opening rescues the reduction of mitochondrial transmembrane potential and impairment of mitochondrial respiration in *PINK1*−/− cells

**Figure 5:**
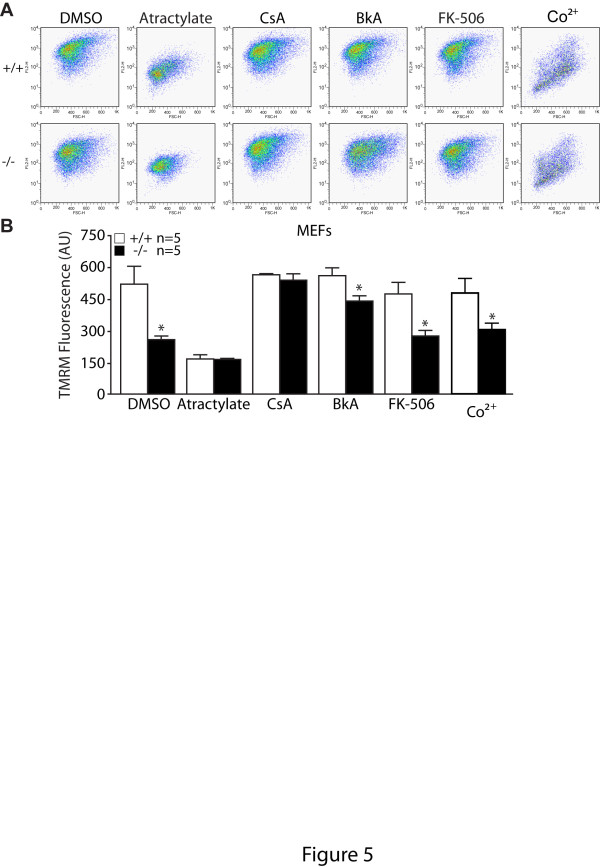
**Blockade of mPTP opening attenuates the defect in mitochondrial transmembrane potential in*PINK1*−/− MEFs. A**. Representative flow cytometry dot plots showing TMRM fluorescence in *PINK1*−/− and +/+ cells incubated with TMRM (50 nM) and atractylate (20 μM), CsA (1 μM), BkA (10 μM), FK-506 (5 μM) or Co^2+^ (1 mM). **B**. The bar graph shows mitochondrial membrane potential measured by TMRM fluorescence after FACS analysis following various treatment, atractylate (20 μM), CsA (1 μM), BkA (10 μM), FK-506 (5 μM) or Co^2+^ (1 mM). The number shown in the panel indicates the number of independent experiments performed. All data are expressed as mean ± SEM. * *p* < 0.05.

To investigate whether increased opening of mPTP underlies the mitochondrial functional defects in *PINK1*−/− cells, we treated cells with mPTP agonist, atractylate, as a positive control of the effects of mPTP opening on mitochondrial depolarization. Treatment with atractyloside acid results in a 64% reduction of TMRM fluorescence in wild-type control cells compared to basal conditions, indicating a strong depolarization of mitochondrial inner membrane (Figure 5A and 5B).

We then treated the cells with mPTP inhibitors to determine whether inhibition of mPTP opening can rescue the transmembrane potential deficits in *PINK1*−/− cells. Indeed CsA treatment fully rescued the reduced TMRM fluorescence in *PINK1*−/− MEFs (Figure 5). Bongkrekic acid also partially rescued the reduced TMRM fluorescence in *PINK1*−/− MEFs (Figure 5). FK-506 treatment had no effect on TMRM fluorescence (Figure 5). Furthermore, cobalt treatment did not alter TMRM signals in *PINK1*−/− and +/+ MEFs, indicating that cobalt does not affect mitochondrial membrane potential in our experimental time frames (Figure 5). These results show that inhibition of mPTP opening rescues the reduction in mitochondrial transmembrane potential in the absence of PINK1, suggesting that increased opening of the mPTP underlies reduction of ΔΨm.

**Figure 6:**
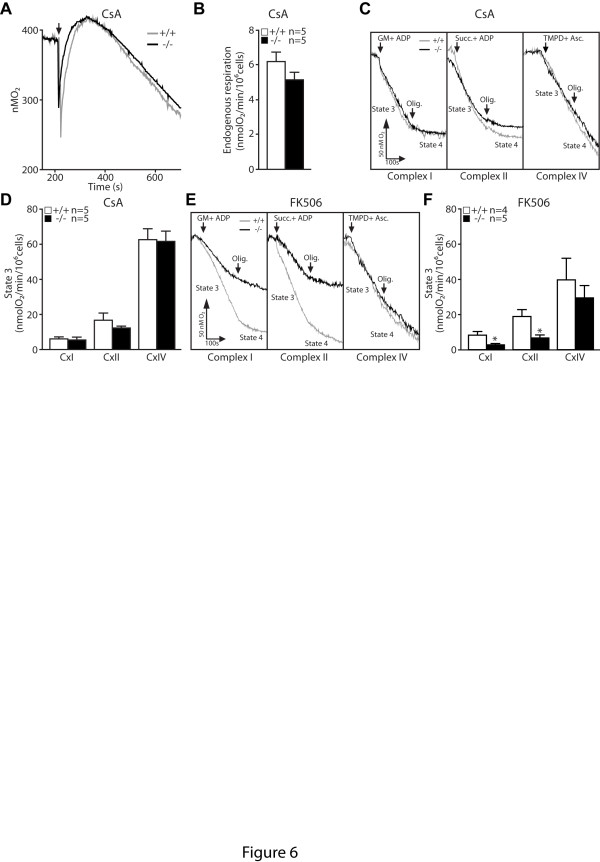
**Blockade of mPTP opening by CsA attenuates the respiratory defect in*PINK1*−/− MEFs. A**. Representative oxygraphs of *PINK1*−/− and +/+ MEFs energized with glucose (10 mM) in the presence of CsA (1 μM). The arrows indicate the time MEFs are added to the chamber. **B**. Oxygen consumption, which represents the endogenous respiratory activity in *PINK1*−/− and +/+ MEFs after treatment with CsA (1 μM). **C**. Representative oxygraphs of *PINK1*−/− and +/+ MEFs energized with 10 mM glutamate/malate (complex I substrate), 10 mM succinate (complex II substrate) or 1 mM TMPD/1 mM ascorbate (complex IV substrate) in the presence of CsA (1 μM). Arrows indicate the time of the addition of either the substrate or oligomycin (2 μM). **D**. Graph showing State 3 respiratory activity for complex I, complex II and complex IV in *PINK1*−/− and +/+ MEFs permeabilized with digitonin after treatment with CsA (1 μM). **E**. Representative oxygraphs of *PINK1*−/− and +/+ MEFs energized with 10 mM glutamate/malate (complex I substrate), 10 mM succinate (complex II substrate) or 1 mM TMPD/1 mM ascorbate (complex IV substrate) after treatment with FK-506 (5 μM). Arrows indicate the time of the addition of either the substrate or oligomycin (2 μM). **F**. Graph showing State 3 respiratory activity for complex I, complex II and complex IV in *PINK1*−/− and +/+ MEFs permeabilized with digitonin after treatment with FK-506 (5 μM). All data are expressed as mean ± SEM. * *p* < 0.05.

We further tested whether inhibition of mPTP opening reverses the mitochondrial respiration impairment in *PINK1*−/− cells. Treatment with CsA reduced the genotypic difference between *PINK1*−/− and control cells to the extent that endogenous respiratory activity is similar (Figure 6A and 6B). Moreover, CsA treatment almost fully rescued complex I driven respiration in *PINK1*−/− cells (Figure 6C and 6D). Treatment with FK-506 was not able to rescue the respiratory impairment in *PINK1−/−* cells, indicating that the effect of CsA on respiration was specific for its inhibitory effect on mPTP (Figure 6E and 6F).

### Normal levels of oxidative stress in *PINK1*−/− cells

**Figure 7:**
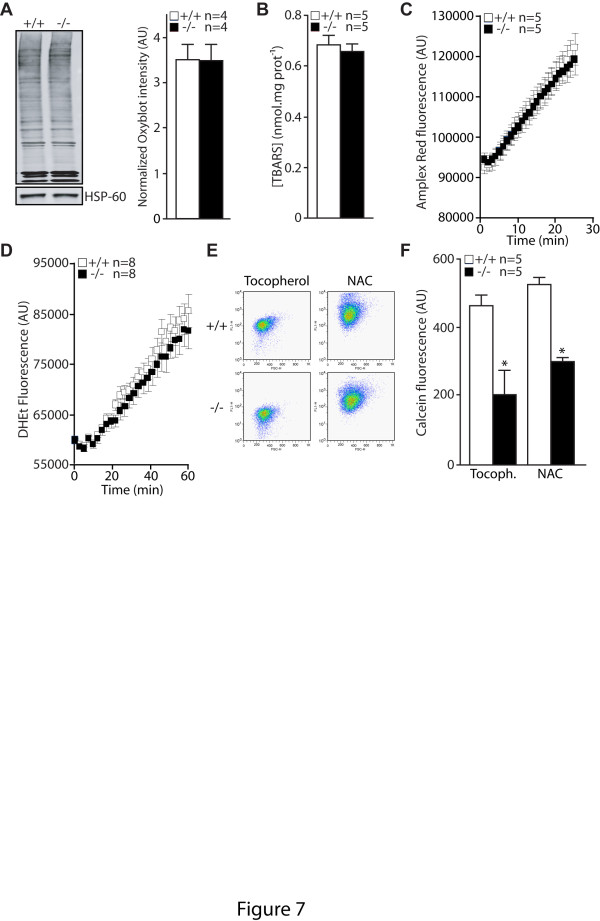
**Normal levels of oxidative stress markers and normal production of reactive oxygen species in*PINK1*−/− MEFs. A**. Left panel: OxyBlot analysis of mitochondrial fractions showing levels of protein carbonyls in both genotypes. Right panel: Quantification of levels of protein carbonyls in *PINK1*−/− and +/+ MEFs. **B**. Levels of lipid peroxidation measured by the TBARS assay in *PINK1*−/− and +/+ MEFs. **C**. Kinetics of H_2_O_2_ production in isolated mitochondria measured by following Amplex Red fluorescence over time. **D**. Kinetics of cytosolic superoxide anion O_2_^-^ production measured in resuspended MEFs by following DHEt fluorescence over time. **E**. Representative flow cytometry dot plots showing calcein fluorescence in *PINK1*−/− and +/+ MEFs in the presence of Co^2+^ after antioxidant treatment with Tocopherol (50 μM, 4 hr) or NAC (1 mM, 2 hr). **F**. Quantification of calcein fluorescence in *PINK1*−/− and +/+ MEFs following treatment with Tocopherol (50 μM, 4 hr) or NAC (1 mM, 2 hr). All data are expressed as mean ± SEM. *p < 0.05.

Because mPTP opening can be affected by elevated oxidative stress [29], we went further to examine whether there is an accumulation of oxidative species in the mitochondrial fraction of *PINK1*−/− and control MEFs. We measured the levels of protein carbonyls, a marker of protein oxidation. As measured by OxyBlot, the total level of carbonyls is similar between the two genotypic groups (Figure 7A). We then measured the accumulation of another common marker of oxidative stress, thiobarbituric acid reactive substances (TBARS), which reflects lipid peroxidation, and found no significant differences between the two genotypes (Figure 7B). We further evaluated the production of oxidative species. Using the Amplex Red dye fluorescence assay we evaluated the propensity of cells to generate Reactive Oxygen Species (ROS) by measuring the production of H_2_O_2_. Because H_2_O_2_ extrusion across the plasma membrane can be kinetically limiting we measured the rate of H_2_O_2_ produced by isolated mitochondria from MEFs. Mitochondria are the main source of ROS in the cells. We found that isolated mitochondria from *PINK1*−/− and WT cells energized with succinate (10 mM) produce H_2_O_2_ at similar rates (Figure 7C). We also monitored the production of superoxide anion O_2_^**.**-^_._ Superoxide is the primary oxidant species generated as a byproduct of mitochondrial respiration. Using the DHEt dye fluorescence assay, we found similar kinetics of O_2_^**.**-^ generation between *PINK1*−/− and WT MEFs (Figure 7D). As positive controls we used MEFs derived from our *DJ-1*−/− mice. Using the same assay conditions, DJ-1 MEFs displayed higher rates of H_2_O_2_ and O_2_^**.**-^ production as monitored with the Amplex Red and the DHEt assays (data not shown). Thus, loss of PINK1 does not increase the production of reactive oxygen species.

We further evaluated the effect of antioxidant treatments on mPTP opening. Cells were treated with Tocopherol and N-acetyl-Cysteine. Tocopherol is a cell permeable antioxidant, which reduces lipid oxidative stress and has been shown to prevent mPTP opening in conditions of elevated oxidative stress [38]. N-Acetyl-Cysteine is a cell permeable precursor of glutathione, which can prevent oxidative stress induced mPTP [39]. Calcein fluorescence levels remained reduced in *PINK1*−/− MEFs compared to control cells following antioxidant treatment (Figure 7E and 7F). These results suggest that increased mPTP opening in *PINK1*−/− MEFs unlikely results from elevated oxidative stress.

### Normal mitochondrial morphology in *PINK1*-deficient cells

**Figure 8:**
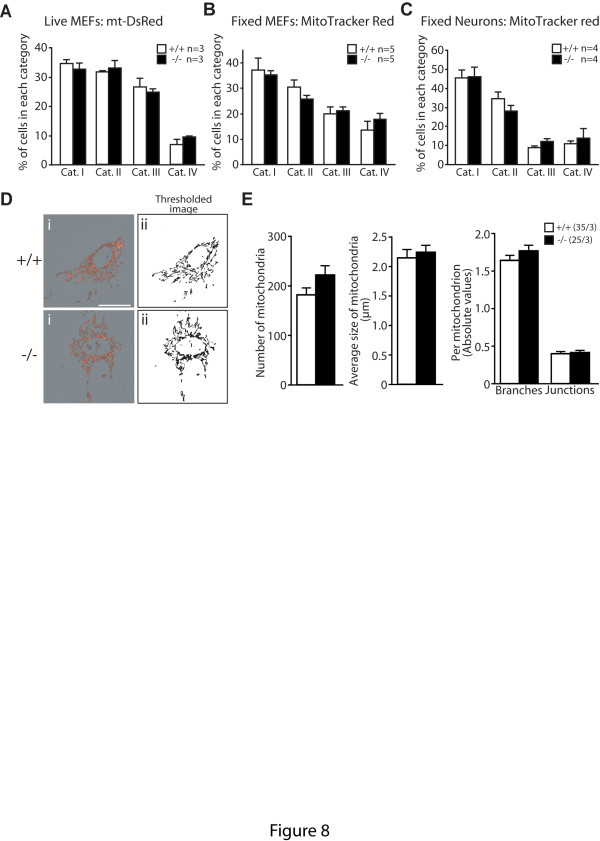
**Unaltered mitochondrial morphology in*PINK1*−/− MEFs and neurons. A**. Distribution of live *PINK1*−/− and +/+ MEFs infected with Mt-DsRed to the four morphological categories I, II, III and IV. **B**. Distribution of fixed *PINK1*−/− and +/+ MEFs stained with MitoTracker Red (250 nM) to the four morphological categories. **C**. Distribution of fixed *PINK1*−/− and +/+ cortical neurons stained with MitoTracker Red (100 nM) to the four morphological categories. **D**. Representative fluorescent images of mitochondria stained with MitoTracker Red (250 nM) i) and the resulting binary (ii) used for quantitative analysis. **E**. Quantitative analysis using ImageJ shows the size and the number of branches of mitochondria in *PINK1*−/− and +/+ MEFs (*P* > 0.05 for all parameters measured). All data are expressed as mean ± SEM. The numbers shown indicate the number of independent experiments performed in the study. For each experiment 100 cells were analyzed quantitatively.

Given the earlier reports on the effects of PINK1 inactivation in mitochondrial fusion [9,40,41] and fission [10,13,42] in fruit flies and mammalian cell lines, we examined whether mitochondrial morphology is affected in *PINK1*−/− MEFs and neurons, which could contribute to the functional deficits we observed in these cells. We assessed mitochondrial morphology in *PINK1*−/− and wild-type MEFs using a previously established method [25]. MEFs were transfected with the mitochondria targeted fluorescent protein mt-dsRed then analyzed by distributing cells into four different categories according to the morphology of their mitochondrial network: (I) cells with a tubular mitochondrial network and less than 5 small round mitochondria; (II) cells with more than 50% tubular mitochondria; (III) cells with more than 50% small and spherical mitochondria; (IV) cells with a fragmented mitochondrial network and less than 5 tubular mitochondria. Under basal conditions, *PINK1*−/− and WT MEFs show similar distribution among the four categories (Figure 8A). To circumvent any possible artifacts due to overexpression of a mitochondrial protein, we performed the same analysis using MitoTracker Red on fixed cells and still did not observe any significant differences between the two genotypic groups (Figure 8B). A similar study on primary cortical neurons derived from *PINK1*−/− and WT mice also showed comparable tubularity of the mitochondrial network in both genotypic groups (Figure 8C).

We next sought to assess mitochondrial morphology in a more quantitative manner. We quantified the number and the average size of mitochondria in each cell using ImageJ on binary images (Figure 8D panels i and ii) of MitoTracker Red stained cells as previously described [26]. We did not observe significant differences between genotypes in these two parameters (Figure 8E). We further quantified the number of branches and junctions per mitochondrion using mitochondrial skeleton images, and did not observe any significant differences between the genotypes (Figure 8E). Thus, loss of PINK1 does not affect mitochondrial morphology in primary cultured MEFs and neurons, at least under our culture conditions. These results suggest that the functional deficits observed in *PINK1*−/− MEFs and neurons are unlikely due to altered mitochondrial morphology.

### Increased mitochondrial calcium concentrations in *PINK1*-deficient cells

**Figure 9:**
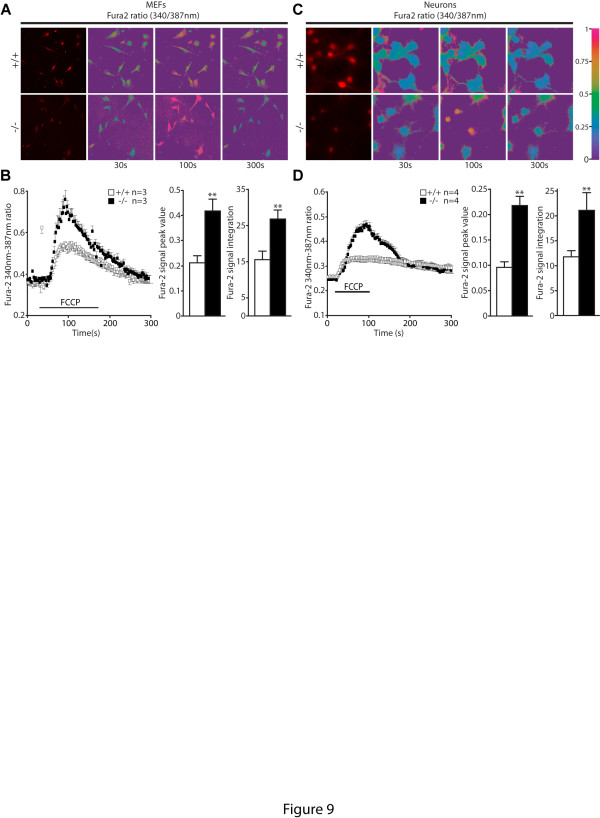
**Higher mitochondrial calcium levels in*PINK1*−/− cells. A**. Representative Fura-2 images of Ca^2+^ responses following FCCP treatment in *PINK1*−/− and +/+ MEFs. Fura-2 ratios at 340/387 are shown at time points indicated. The pseudocolor calibration scale for 340/387 ratios is shown on the right. FCCP (1 μM) was added at t = 25 s. **B**. Left panel: Time course of cytosolic [Ca^2+^] rise following FCCP treatment in *PINK1*−/− and +/+ MEFs. Middle panel: The peak value of cytosolic calcium rise following FCCP is higher in *PINK1*−/− cells. Right panel: The average size of the FCCP releasable Ca^2+^ pool is higher in *PINK1*−/− MEFs compared to controls. **C**. Representative Fura-2 images of Ca^2+^ responses following FCCP treatment in *PINK1*−/− and +/+ neurons. **D**. Left panel: Time course of cytosolic [Ca^2+^] rise following FCCP treatment in *PINK1*−/− and +/+ neurons. Middle panel: The peak value of cytosolic calcium rise following FCCP is higher in *PINK1*−/− neurons. Right panel: The average size of the FCCP releasable Ca^2+^ pool is higher in *PINK1*−/− neurons. All data are expressed as mean ± SEM. ** *p* < 0.01.

We also evaluated mitochondrial calcium in the absence of PINK1, since elevated mitochondrial calcium levels are known to induce the opening of the mPTP [43]. We measured the size of the mitochondrial calcium pool by quantifying the amount of calcium released from mitochondria to the cytosol following FCCP treatment (1 μM). FCCP is a specific proton ionophore that dissipates proton gradients and allows cations to freely redistribute across membranes according to their concentration gradients. Following FCCP treatment, alterations of cytosolic calcium concentrations were monitored with Fura-2, a BAPTA based dye whose fluorescence excitation ratio at 340 nm and 387 nm are proportional to cytosolic calcium concentrations. Increases in Fura-2 signals following FCCP treatment are much higher in *PINK1*−/− MEFs (Figure 9A and 9B) and neurons (Figure 9C and 9D) compared to their respective wild-type controls. These results suggest loss of PINK1 results in selective increase in mitochondrial calcium.

## Discussion

In the current study, we investigated the mechanism underlying the mitochondrial respiration defects caused by loss of PINK1. We established primary MEFs and cortical neuronal cultures from our *PINK1*−/− mice to evaluate mitochondrial functions in intact cells. Similar to what we previously reported in mitochondria isolated from mouse brains [18], mitochondrial respiration is impaired in *PINK1*−/− MEFs, indicating that these cells represent a valid cellular model to study the detailed mechanisms underlying respiratory defects seen in *PINK1*−/− mice (Figure 1). Although respiration impairment can be caused by defects in mitochondrial transmembrane potential or the electron transport system, we found that only the mitochondrial transmembrane potential is reduced in *PINK1*−/− cells (Figure 2), while enzymatic activities of the complexes composing the electron transfer system are unaffected (Figure 1). In search for mechanisms underlying the reduction of the transmembrane potential, we found that opening of the mitochondrial permeability transition pore is increased in the absence of PINK1 and that this defect can be rescued by inhibitors of the mPTP (Figures 3 and 4). Furthermore, mitochondrial transmembrane potential and respiration defects caused by loss of PINK1 were also reversed specifically by inhibitors of the mPTP (Figures 5 and 6), suggesting that increased opening of the mPTP underlies the defects in mitochondrial transmembrane potential and respiration observed in *PINK1*−/− cells. These mitochondrial functional defects occur in the absence of elevated oxidative stress (Figure 7) and mitochondrial morphological changes (Figure 8), but mitochondrial calcium is increased in *PINK1*−/− cells, suggesting that elevated mitochondrial calcium underlies the increase in mPTP opening (Figure 9).

Following our initial report of mitochondrial respiration defects in *PINK1*−/− mouse brains [18], a growing consensus has been building on the importance of PINK1 in mitochondrial respiration [15-19,44], though the underlying mechanism remained unclear. Defects in the activity of the electron transport system complexes have been suggested as a possible mechanism underlying the respiratory defects resulting from the loss of PINK1, as silencing PINK1 expression by siRNA in SH-SY5Y cells affected mitochondrial ATP synthesis and activity of ETC complexes [19]. However, enzymatic activities of the ETC complexes in our primary *PINK1*-deficient cells are normal. Instead we found that loss of PINK1 increased opening of the mitochondrial permeability transition pore, and that blocking mPTP opening occluded the difference between *PINK1*−/− and control MEFs for endogenous and State 3 respiration. These results suggest that increased mPTP opening is responsible for reduced mitochondrial respiration in *PINK1*−/− cells. Previous reports showed that mPTP opening triggered by elevated calcium concentrations leads to reduced state 3 respiratory activities [45,46], an effect that can be prevented by pretreatment with CsA [45,47,48].

Reduced transmembrane potential in *PINK1*-deficient cells has been reported in a wide variety of cells [15-17,21,49]. In accordance with these previous reports, we also found that ΔΨm is reduced in primary *PINK1*−/− fibroblasts and neurons. It has been proposed that reduced enzymatic capacity of complex I of the mitochondrial electron transport system might be the underlying cause of the defects in ΔΨm [11]. However, similar to other previous studies [17,50,51], we found that complex I enzymatic activity as well as the activity of all other complexes composing the ETS are normal in our cell models in the absence of PINK1. Rather, we found that increased opening of the mPTP likely plays an important role in the mitochondrial depolarization observed in *PINK1*−/− cells, as inhibitors of the mPTP, CsA and BkA, rescued the ΔΨm defects in *PINK1*−/− cells. The opening of the mPTP allows free diffusion of small ions across the mitochondrial inner membrane as a corrective mechanism for cation overload [52-54]. Hence, increased opening of the mPTP allows a partial depolarization of the mitochondrial membrane, and this defect can be reversed by inhibition of mPTP opening such as CsA [24,52]. The stronger rescuing effect observed with CsA might relate to the fact that, in addition to blocking the mPTP, it also hampers mitochondrial calcium uptake, which may be increased in *PINK1*-deficient cells, as suggested by higher mitochondrial calcium levels in these cells.

A possible role of PINK1 in modulating mitochondrial morphology and dynamics emerged from studies in *Drosophila*. Loss of PINK1 function in flies results in abnormally large mitochondria with fragmented cristae and reduced capacity to generate ATP [6,7]. This mitochondrial phenotype is suppressed by genetically promoting mitochondrial fission or decreasing mitochondrial fusion, inferring that perturbed mitochondrial fission in *PINK1*-deficient models underlies functional defects [9,40,41]. However, whether and how PINK1 may regulate mitochondrial morphology and dynamics in mammalian cells is much less clear. The effects of PINK1 deficiency on mitochondrial morphology and dynamics seem to depend on the cell type studied and range from inducing mitochondrial fission [10,42] or fusion [14] to no effect [11,16,19,50]. Consistent with these studies [11,16,19,50], our analysis of primary cultured *PINK1*−/− MEFs and neurons did not show overt changes in mitochondrial morphology in fixed or live cells (Figure 8). These findings are also in agreement with our earlier EM study showing that no drastic ultrastructural changes in mitochondrial number and integrity in *PINK1*−/− brains at 3–4 and 22–24 months of age [18]. Thus, loss of PINK1 function causes mitochondrial functional defects, in the absence of morphological changes, suggesting that the morphological abnormalities observed in mammalian cell lines may be downstream consequences of these mitochondrial functional defects.

How does loss of PINK1 lead to increased mPTP opening? Opening of the mPTP is primarily induced by oxidative stress and/or elevated intramitochondrial calcium concentrations [45]. We did not find any evidence of oxidative damage or increased production of ROS in *PINK1*-deficient cells (Figure 7). However, we found that mitochondrial calcium concentration measured indirectly in the cytosol following FCCP treatment is increased in *PINK1*−/− MEFs and neurons (Figure 9). This observation is consistent with a recent study showing that loss of PINK1 reduces the activity of the mitochondrial Na^+^/Ca^2+^ exchanger (NCX), which regulates Na^+^-dependent Ca^2+^ efflux [15]. Pharmacologic inhibition of NCX activity leads to accumulation of calcium in isolated mitochondria [55]. It is therefore possible that impaired NCX activity in *PINK1*−/− cells may lead to accumulation of mitochondrial calcium, which in turn increases the opening of the mPTP. In this context, the mPTP may serve as a Ca^2+^-activated Ca^2+^ release channel [56]. However, it is unclear how loss of PINK1 affects the activity of the mitochondrial Na^+^/Ca^2+^ exchanger. A direct regulation of the mitochondrial Na^+^/Ca^2+^ exchanger by PINK1-mediated phosphorylation is possible but difficult to demonstrate, since the molecular nature of the Na^+^/Ca^2+^ exchanger is unknown.

Mitochondrial dysfunction has long been thought to play an important role in the pathogenesis of Parkinson’s disease [57]. This is based on earlier studies using postmortem idiopathic PD brains showing mitochondrial respiration impairment and oxidative damage [3], and on findings that mitochondrial complex I inhibitors, such as MPTP and rotenone, produce parkinsonian syndromes in humans and experimental animal models [4,5]. Our prior reports showing mitochondrial respiration defects in *Parkin*−/− and *PINK1*−/− mouse brains and linking these recessive PD genes to mitochondrial function provided experimental evidence in support of a causal role of mitochondrial functional impairment in PD pathogenesis [18,58]. The current study highlights the importance of mitochondrial permeability transition pore opening in PINK1 mediated mitochondrial respiration and function. Our recent unpublished work further showed that loss of Parkin or DJ-1 also leads to increases in mPTP opening (EG and JS, unpublished data). Thus, increased mPTP may be a common mechanism leading to PD pathogenesis.

In summary, our study highlights an important role of PINK1 in the regulation of mitochondrial permeability transition pore. Our findings suggest that dysregulation of the opening of the mPTP likely underlies impairment of mitochondrial respiration and reduction of mitochondrial transmembrane potential. Future studies will be needed to elucidate the mechanism by which PINK1 regulates mitochondrial calcium homeostasis and opening of the mPTP. Given the importance of mPTP opening in the regulation of the release of proapoptotic factors from mitochondria to the cytosol, it will be important to determine whether alteration of mPTP opening is a key mechanism underlying increased vulnerability of *PINK1*-deficient cells to exogenous stressors [11,59]. In addition, future studies are needed to determine whether increased opening of the mPTP is a feature common to other genetic forms of the disease, and whether modulation of its opening may provide a novel therapeutic strategy for the treatment of Parkinson’s disease.

## Competing interests

The authors declare that they have no competing interests.

## Authors’ contribution

CG and JS conceived and designed the study and wrote the manuscript. CG and EG carried out the experiments and obtained the data for Figures 1-9. LM, EC and CV participated in experimental design for Figure 9, and ZS and DC carried out the mitochondrial morphological analysis using the retroviral vectors mt-DsRed in Figure 8. All authors read and approved the final manuscript.
